# Considerations in Surgical Convalescence

**Published:** 1910-09

**Authors:** H. P. Cole

**Affiliations:** Mobile, Ala.


### CONSIDERATIONS IN SURGICAL CONVALESCENCE.

By H. P. Cole, M. D., Mobile, Ala.

Practical application of modern discoveries in bacteriology, pathology and physiology to the surgical treatment of human ills, has marked a triumphant invasion of all portions of the human organism. Surgical success of the past four decades has stimulated a warranted enthusiasm in surgical science and has produced innumerable operators and a few great surgeons.

One of the teachers has well said, "Common sense in matters medical is rare, and is usually in inverse proportion to the degree of education." Natural sagacity frequently determines the difference between the surgeon and the operator, and the application of good sound judgment to the management of surgical convalescence has accomplished more in that than in any other department of surgery.

It is only in recent years that the convalescence has received intelligent and sober consideration. There are in existence today numerous recent publications dealing with post operative treatment and no modern surgical treatise is now complete without its chapter treating of operative sequelae. However, as in many another branch, its exhaustive portrayal by the pen of most writers has only served "to make confusion worse confounded." Frequently the scientific has been achieved, but to the detriment of the practical.

Admitting the value of scientific application of modern discoveries, we decry the tendency to overshadow good sense with an over application of scientific principles. It is the object of this brief essay to plead the application of sound judgment in the treatment of surgical sequenlae, as opposed to the all too prevalent dogmatic practices resulting from the over application of the results of modern researches.

How many of us have fallen into a mechanical or automatic method of post-operative treatment, a treatment applied to patients in general rather than to the needs of patients in particular? Permit us to recall a few of our old familiar friends; repeated doses of morphine, strychnine, sparteine, esserine and the like—any or all ordered for the same patient. How frequently are these anticipatory and unnecessary rather than indicated for actual relief of conditions present? How often might we note in our post-operative orders such dogmata as these, "the patient to have nothing by mouth 'til nausea ceases," "the patient to be catheterized in 8 hours," "the patient to have calomel and soda on the evening of the second day?" How often do we institute a "foodless food" regime of lemon albumen, beef tea and kindred valueless liquids? In how many clinics might we find dogmatic rules regulating the very day upon which a patient may be turned, may have a back-rest, sit in a chair and so on, to a prescription of the actual day upon which he may leave the hospital, a day ordained and prescribed before the patient has even entered the hospital? Mechanical rules better suited to the handling of manikins than to the treatmet of mankind.

In applying rational therapy to post-operative sequelae let us first consider post-operative pain. Pain in the early hours frequently results from an unnatural or awkward position. The abdominal binder may need adjustment, not infrequently a distended bladder is the source of an apparently inexplicable abdominal pain. Searching for and eliminating these factors frequently obviates the use of morphine. A competent nurse who understands the value of the ice and hot water bags will frequently accomplish wonders in the relief of this early post operative feature.

A pillow placed beneath the knees of a patient operated on in the dorsal position, flexes the thighs and obviates back pains resulting from over stretching of the psoas muscles. Not infrequently the patient is carried comfortably through the early painful period by the administration of morphine and atropine during or immediately following the operation. The nature of the operation and the peculiarities of the patient's disposition should determine the necessity of its administration. Its early use frequently obviates a second or repeated dosage, undesirable in many cases because of complications ensuing from its frequent admistration.

Delayed abdominal pain, usually due to distension, is usually relieved by the high insertion of a rectal tube, together with turning of the patient from side to side, permitting of free expulsion of gases.

Thirst, nausea and vomiting are frequently allayed through the adminstration of slow saline enemata. Water may usually be given by mouth in satisfying amounts in the very cases where it is common practice to withhold it till nausea ceases. If vomited, the water soon serves to eliminate the nausea producing ether which is secreted in the stomach, Application of ice-bags to the throat and epigastrium are simple methods of allaying nausea and thirst.

Mechanical maneuvers, such as elevation of the patient's head or trunk, placing in a sitting position, turning upon one or the other side or occasionally upon the abdomen, frequently serve to arrest early persistent nausea and vomiting.

Nausea and vomiting are frequently obviated in selected cases by gastric lavage before recovery from the anaesthetic. The early administration of morphine and atropine in selected cases serves to relieve rather than produce nausea and vomiting.

Thirst, nausea and vomiting today should not be the serious post-operative features of former times, if one follows modern methods of anaesthesia. The preliminary use of nitrous oxide, or ethyl chloride, the open air method of ether administration and frequently the administration of oxygen following, tend to eliminate thirst, nausea and vomiting as distressing operative sequelae.

Insomnia and nervousness as occasional sequelae are frequently due to an uncomfortable position occupied by the patient. Undue apprehension when relieved by tactful assurance frequently results in the dissipation of insomnia and nervousness. The exclusion of solicitous relatives and friends during the early hours following the operation frequently serves to forestall these features.

No one feature of the convalescence has given rise to more concern and dogmatic practice than has evacuation of the bowels, to-wit:—routine administration of eserine, calomel, castor oil and similar cathartics. Many cases if properly prepared before and properly nourished after operation, experience a normal evacuation of the bowels on the third or fourth day. At most a small soapsuds enema or the rectal adminstration of salts or glycerine on the third day, usually suffices, and this without the early, painful and often deleterious peristalsis resulting from fear inspired drastic and routine catharsis.

Evacuation of the bladder is frequently voluntary if not obviated through a standing order to catheterize every 8 to 10 hours.

The application of hot water bags to the lower abdomen and spine, hot rectal lavage, a hot bed-pan or urinal and such maneuveres should be tried before resorting to catheterization. In many cases a change in posture, especially in males, some even being permitted to stand, may allow if micturition and obviate a possible cystitis from catheterization.

Post operative feeding has given rise to many complicated and useless dieting methods. Most abdominal cases tolerate semi-solid food as well or better than liquids shortly after operation.

A normal strength-giving diet is preferable in most patients after there has been a good exacuation of the bowels. In the matter of feeding due care must be observed in selecting food to which the patient has been accustomed before operation, and within reason placing him on his accustomed diet, usually a more strengthening practice than forcing upon a jaded palate food to which it is unaccustomed.

Lastly, there is probably no greater opportuntiy for exercising good judgment than in the consideration of the length of the patients hospital residence. The nature of the operation, the patient's individual peculiarities and his physical and financial condition are the factors upon which we must determine his needs for long or short confinement to bed and hospital.

Aged patients are susceptible to static complications following protracted confinement to bed. The excellent results from prostatectomy in aged patients in the last few years have in large part resulted from their early removal from bed. Rural patients suffer from prolonged bed and hospital confinement as do a certain type of neurotics. Many of these, financially able to occupy private rooms, may well be placed in the open ward permitting of constant companionship. A certain type of neurotic patient develops the hospital habit if permitted a long hospital residence and the seclusion of the private room. A short period in bed and hospital and all they imply to this type will result in a greater per cent. of permanent cure.

The financial status of a majority of patients demands a return to earning capacity at the earliest date consistent with a safe convalescence.

There are undoubtedly a host of enthusiasts who are too early removed from both bed and hospital, yet we believe that there is a too prevalent custom of allowing prolonged routine treatment as to the residence without adequate study of individual necessity for early removal.

The opponents of shortening the average time of bed postural treatment advance two principal objections: an increased liability of hernia, and of thrombosis and embolism. The statistics available from hundreds of abdominal sections allowed in a chair in from 1 to 4 days after operation fail to show any increased liability to hernia. A proper and snugly fitting abdominal binder so splints the abdominal muscles as to obviate the possibility of hernia formation. Likewise there is no evidence to indicate any increased liability to thrombosis and embolism, in fact, there is considerable ground to feel that the liability is lessened by a shorter bed and hospital convalescence.

We have allowed a large number of suitable clean laparotomy patients in a chair within from 1 to 5 days after operation. Many walked on the third or fourth day. We have observed no ill-effects and may claim the following advantages of this procedure:

1. There is a mental stimulus to a large class of neurotic patients, a large factor in the permanence of cure.

2. There is an earlier return of vascular tone, an earlier desire for food, with a resultant early return to strength. Thus there is an appreciable shortening of the hospital residence, from 7 to 10 days in many cases. Likewise this results in a financial saving to a large class of cases, both through lessened hospital expense and through an earlier return to earning capacity.

3. The early period of convalescence is frequently more easily born, from the comfort of the siting posture, early liberty of movements and earlier and more frequently normal bowel evacuation.

In concusion we plead for the application of personal knowledge of the individual patient as opposed to the general routine in the management of surgical convalescence.

